# Inequities in coverage of smokefree outdoor space policies within the United States: school grounds and playgrounds

**DOI:** 10.1186/s12889-018-5602-7

**Published:** 2018-06-14

**Authors:** Christopher Lowrie, Amber L. Pearson, George Thomson

**Affiliations:** 1 Department of Geography, Environment, and Spatial Sciences, Michigan State University, 673 Auditorium Road, East Lansing, MI 48824 USA; 2 Department of Public Health, University of Otago, Wellington, 6021 New Zealand; 3 Environmental Science and Policy Program, Michigan State University, East Lansing, MI 48824 USA

**Keywords:** Smoking, Inequalities, Smokefree outdoor spaces, Geography

## Abstract

**Background:**

Previous studies have found extensive geographic and demographic differences in tobacco use. These differences have been found to be reduced by effective public policies, including banning smoking in public spaces. Smokefree outdoor spaces reduce secondhand smoke exposure and de-normalize smoking.

**Methods:**

After previously publishing a study of smokefree indoor and outdoor space policies, it was brought to the authors’ attention that the dataset used in analyses was incomplete (Lowrie et al., BMC Public Health 17:456, 2017). The current manuscript is a corrected version. Here, we include analyses for outdoor space policies. We evaluated regional and demographic differences in the proportion of the population (both adult and child) covered by smokefree outdoor space policies for school grounds and playgrounds enacted in the United States prior to 2014.

**Results:**

Children had a low level of protection in playgrounds and schools (8% covered nationwide in both settings). Significant differences in coverage were found by ethnicity, region, income, and education (*p* < 0.001). The odds of having a smokefree playgrounds policy was lower for jurisdictions with higher proportions of poor households, households with no high school diploma, whites and the Alaska/Hawaii region. Increased ethnic heterogeneity was found to be a significant predictor of increased odds of having a smokefree playgrounds policy, meaning that diversity is protective, with differential effect by region (*p* < 0.001) – which may relate to urbanicity.

**Conclusions:**

Disparities in smokefree outdoor space policies have potential to exacerbate existing health inequities. A national increase in smokefree outdoor space policies to protect children in playgrounds and schools is a crucial intervention to reduce such inequities.

## Background

Tobacco use continues to be a primary global health issue, with over 180 nations committed to reducing smoking as signatories to the Framework Convention on Tobacco Control [1]. Cigarette smokers have shorter lifespans than non-smokers by at least 10 years [2], due to many health issues, including cancer, cardiovascular diseases, and respiratory diseases [3]. The negative effects of cigarette smoking are large and well-documented for most countries. In the United States, more people have been prematurely killed by cigarette smoke than in all of the nation’s wars combined [2]. In the United Kingdom, 19% of cancer cases are linked with exposure to cigarette smoke [4]. And in China, alone, approximately 1 million deaths are linked to cigarette smoke each year [5]. Globally, tobacco kills more than 6 million people each year [1].

These impacts are distributed unequally, with consequent inequities. For this work, we adapt a definition of inequalities as ‘the different availability of resources to which individuals and groups have access to’ [6]. A range of studies have found evidence of inequities in tobacco smoking and secondhand smoke (SHS) exposure [5, 7, 8], with minorities generally experiencing higher risks related to tobacco. Research in the United States revealed differences in likelihood of smoking by ethnicity, which became larger with age [9]. Another study in the United States found that smokefree home policies were more prevalent in the West, and among those making over $100,000 and those with graduate degrees [10]. A study in the Southeastern United States found that less-educated citizens were less likely to be covered by smokefree space policies, and that smoking was a predictor of dropping out of high school [11]. In addition to ethnic differences, the likelihood of tobacco addiction has been associated with educational attainment, socioeconomic status, and region [5, 11–13]. In a study of American twins in the military, cigarette smoking was found to have an association with lower educational attainment [12]. Geographically, in the United States in 2015 the Midwest had the highest prevalence of cigarettes smoking among adults 18 years and older at 18.7%, above the national prevalence of 15.1%, and the southeastern states of West Virginia and Kentucky had prevalences of 26.7 and 26.2%, respectively [8]. These inequities in smoking prevalences by ethnicity, socioeconomic status, and geography suggest drastically different tobacco related risks within America, dependent on circumstances and experience. To counter this, more widespread policies to cover vulnerable populations are needed.

Research has consistently found that denormalizing smoking is an effective way of decreasing smoking prevalence and preventing initial uptake of tobacco use [14–17]. To denormalize smoking is to reduce its social acceptability and the perception of it as a normal activity, thereby promoting quitting and preventing initiation [18]. Research indicates that interventions aimed at reducing tobacco use are more successful if they change what is considered socially ‘normal’ behavior within the targeted community. Many factors influence the normality of smoking, both positively and negatively. Smoking can be made to seem more normal due to advertisements and efforts by the tobacco industry, which spent $9 billion on advertising in 2014, largely aimed at ethnic minorities [19]. Of importance to the current study, smoking can also be denormalized by intelligent outdoor smokefree space policies. These policies reduce smoking in the public view, and have been shown to deter smoking and increase the perception of smoking as socially unacceptable [20, 21].

One important area of intervention is tobacco use by children, when lifetime use and addiction is typically established [22]. Smokefree space policies protecting places frequented by children are also important for reducing exposure to SHS. Almost half of children worldwide are regularly exposed to SHS in public spaces [1]. Extensive research has found smokefree space policies to be effective in reducing tobacco smoking and increasing cessation, while improving population health and air quality [23]. In California, smoking bans in homes and perceptions of local smokefree outdoor areas were both found to decrease smoking and increase quit attempts [24]. Across the USA, smokefree indoor policy bans were also found to explain smoking prevalence at the state level [25]. In Ontario, exposure to smoking in restaurants and bar patios was found to decrease the likelihood of a quit attempt [26]. Jurisdictions in North America are increasingly adopting legislation for smokefree public spaces; a study in Canada found that indoor smoking restrictions follow a spatial diffusion pattern, with jurisdictions following examples set by nearby and similar jurisdictions [27].

While indoor smoking bans have been widely enacted since 1975 in the United States [28], outdoor smokefree policies are less common. Despite the compilation of a national database on outdoor smokefree space policies in the United States [29] analysis of these important interventions remains limited. We found few existing national studies in the USA on regional differences in smokefree space policies, or on ethnic, socioeconomic, and educational differences in the populations covered by these policies. Studies have been conducted at the state level (e.g. California [30]), and primarily pertain to indoor space policies or have been limited to outdoor spaces such as parks and beaches [31, 32]. In the latter research, the odds of having a local smokefree parks policy was found to increase with higher percentages of Hispanics, people under the age of 18, Democrats, and recent movers; and to decrease with a higher percentage of older voters, smokers, and non-Hispanic Whites [32]. Additionally, lower odds of having a smokefree space policy at the state level was significantly associated with higher percentages of smokers, and rural counties were found to be less likely to have policy protection than urban counties. Using policy data enacted up to 2009, Gonzales et al. found ethnic differences in coverage by indoor smokefree policies in the USA, with Hispanics and Asians having more coverage than Blacks [33]. After previously publishing a study of smokefree indoor and outdoor space policies, it was brought to the authors’ attention that the dataset used in analyses was not complete [34]. The current manuscript is a corrected version and only includes analyses for outdoor space policies.

This research investigated inequalities in coverage by ethnicity, socioeconomic status, educational attainment, and region, for smokefree outdoor space policies for places frequented by children: school grounds and playgrounds. The research questions were: 1) Are there differences in the proportion of the population covered by such smokefree space policies by ethnicity, income, or education?; 2) Are there differences by ethnicity in the proportion of children covered by these policies?; 3) Are there regional differences or geographic patterns in the proportion of the population covered by these policies?; and 4) What is the association between the existence of smokefree playgrounds policies and area-level ethnic heterogeneity, income, educational attainment, percentage white, and region?

## Methods

### Data sources

Tobacco control laws data were provided by the American Nonsmokers’ Rights Foundation U.S. Tobacco Control Laws Database. This database contained zip code (*n* = 1574), county (*n* = 279), and state (*n* = 37) level data for policies enacted pre-2015 covering outdoor public spaces. Explicit in these data were descriptions of the policies and the spaces covered which were used in our analysis. In total, three groups of restrictions were studied: playgrounds, school grounds, and both playgrounds and school grounds. We excluded smokefree policies covering less frequently visited outdoor spaces and places where less time would be spent (e.g., fairs/festivals, public events, and parking lots). State and county policies were applied to all zip codes within the jurisdiction to allow for analysis at the zip code level, resulting in a total of 21,477 zip codes with policies (66% of the US total zipcodes). This builds on the work done and methodology used by Hood et al. [32], in which research was conducted at the county level. By using zip codes, we hoped to obtain a higher resolution of the differences between jurisdictions.

**Fig. 1:**
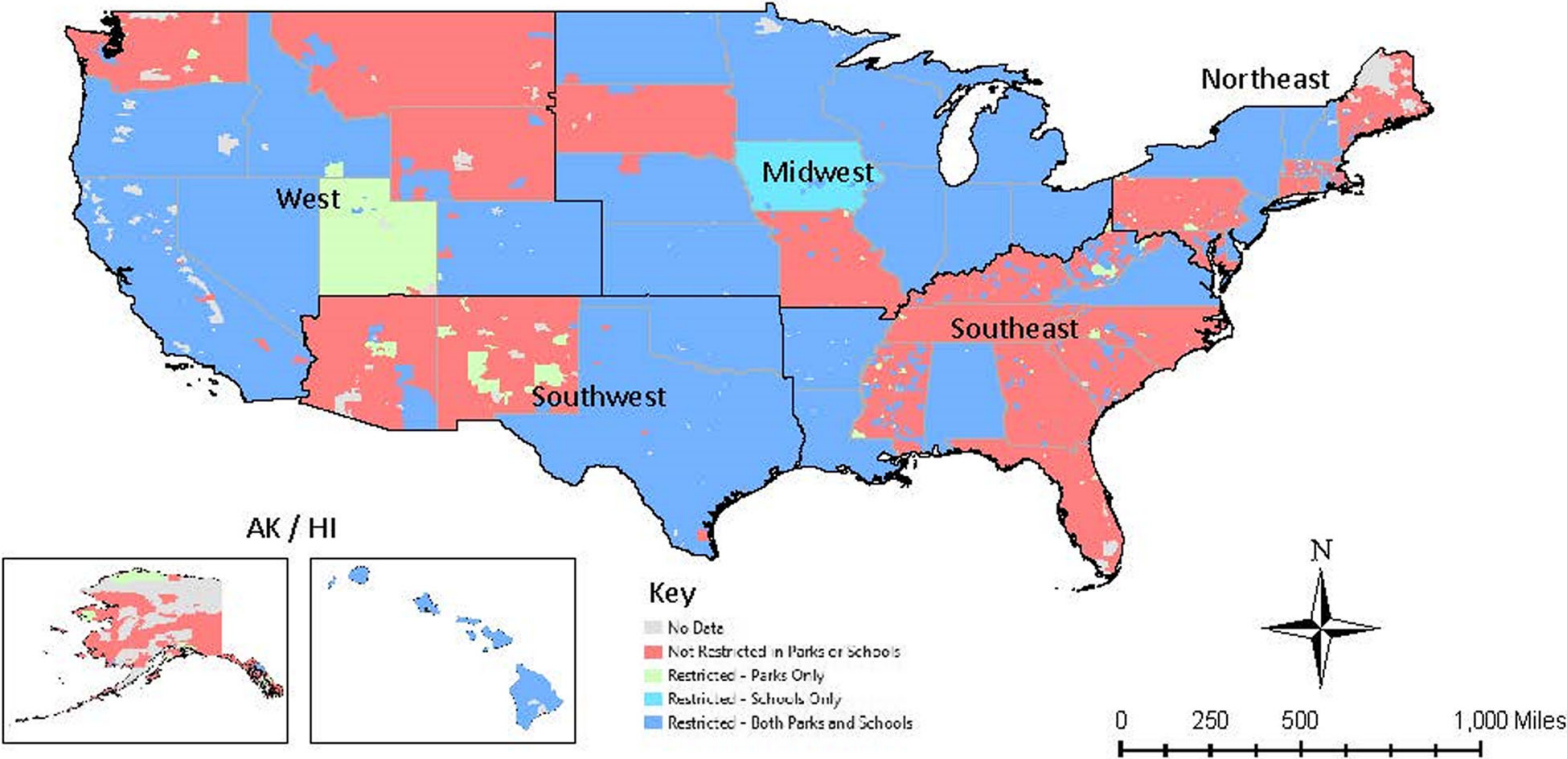
The presence of smokefree outdoor space policies for school grounds, playgrounds, both, or neither by region; map created by the authors

Demographic data were obtained from the US Census (2010) and included age, ethnicity, educational attainment, and income at the zip code level. Income data was used to calculate income quartiles. Each zip code was assigned to one of six regions, based on the state in which it is located (Fig. 1). By doing this, we hoped to improve upon the work done by Gonzalez et al. [32] by updating their 2009 analyses on ethnic inequalities and extending analyses to examine socioeconomic, educational, and regional inequalities. Ethnic heterogeneity was calculated as 1 minus the sum across ethnic groups of their respective squared proportion, as described by Wadsworth and Kubrin [35], and a binary high versus low ethnic heterogeneity variable was created using the mean as the cutoff.

### Statistical and geographic methods

To address the first three research questions, a chi-squared test was used to test for significant differences in counts of population subgroups covered by each of the three types of smokefree outdoor space policies. Analysis was conducted by ethnicity (for the total population and for children <15 years old), income and educational attainment (for adults ≥25 years old), and region (for the total population). To assess geographic patterns in policy adoption, data were imported into ESRI ArcGIS version 10.2 and maps for all zip codes, delineated by region, were created.

To address the fourth research question, we fitted a mixed effects logistic regression model, to test which demographic characteristics were associated with smokefree playgrounds policies. We chose not to fit a model for whether or not a place has a school grounds and playgrounds policy, due to both very low levels of such policies and extreme differences in regions, which would lead to model instability. In our model, the dependent variable of interest was a binary measure of whether each zip code had a policy protecting playgrounds. We also investigated whether interaction terms improved the model fit, using the likelihood ratio test. Specifically, we examined interactions between ethnic heterogeneity and region, and between percentage black and region, and compared the log likelihoods obtained from models including and excluding these interaction parameters. Thus, our final model included the following independent variables: education, high/low ethnic heterogeneity, percentage white, income, and region, as well as an interaction term between ethnic heterogeneity and region. We accounted for non-independence in zip code policy status within state clusters by fitting a hierarchical regression model including random effects at the state level. All statistical analyses were conducted using Stata v14 software.

## Results

Of the approximately 350 million people living in the United States in 2014, only 9% had smokefree outdoor space policy protection for both school grounds and playgrounds within their home zip code (Table 1). Higher proportions of the population were covered under smokefree policies just for playgrounds (28%), with far lower coverage for school grounds (18%). A significant difference was observed between ethnic groups for the proportion covered (*p* < 0.001). Whites followed by Blacks had the lowest proportion of coverage, with only 8 and 9%, respectively, of the population living in zip codes with coverage for both outdoor spaces, and Asians had the highest (13%). The differences were even greater for just playgrounds, with Asian (51%) and Hispanic (40%) coverage much higher than for Whites (23%) and Blacks (22%). The proportions of children covered followed the same trends as the total population, with only 8% of children in the US living in zip codes with smokefree policies for school grounds and playgrounds. We noticed a difference in coverage by wealth, whereby those with the highest income (>$75 k) had a higher proportion of the population covered by these policies (10%) compared to the middle and lowest incomes (8%). These differences were statistically significant (*p* < 0.001). Again, the differences were even greater for just playgrounds, with 29% coverage for the highest income, compared to 22% for the lowest (<$25 k). A significant gradient was found for educational attainment, whereby the highest level of education (graduate degree) had the highest proportion covered by both smokefree space policies (11%) and the lowest levels of education (no high school diploma) had the lowest proportion (7%). There were larger differences for just playgrounds, with 29 and 12% coverages for the highest and lowest education levels.

**Table 1:** Percentage population covered by smokefree school grounds and playgrounds policies, by subgroups

	Playgrounds	School grounds	Both playgrounds and school grounds
Ethnicity - Total population			
*Total*	28	18	9
Asian	51	21	13
Black	22	18	10
Hispanic	40	16	9
White	23	18	8
Other	44	19	12
Ethnicity - Children <15 years			
*Total*	28	17	8
Asian	48	21	12
Black	21	18	10
Hispanic	38	15	7
White	22	18	8
Other	43	18	11
Income^a^			
<$25,000	22	17	8
$25,000–$75,000	23	17	8
>$75,000	29	20	10
Educational Attainment^a^			
No high school diploma	12	19	7
High school diploma	22	18	9
Bachelors	28	20	10
Masters or Doctorate	29	21	11
Region - Total population			
AK/HI	11	~ 0	~ 0
Midwest	7	6	1
Northeast	43	51	39
Southeast	8	10	1
Southwest	3	~ 0	~ 0
West	66	20	2

^a^Calculated for the population over 25 years old

*P*-values calculated using Pearson’s Χ^2^ statistic - all differences *p* < 0.001

By region, the differences were stark. The Northeast had a much higher rate of coverage for smokefree policies for both school grounds *and* playgrounds, compared to the other regions, with 39% of the population covered; no other region had more than 2% of the population covered. Virtually no coverage for both school grounds and playgrounds was observed in AK/HI, and the Southwest. For playgrounds (which tended to have higher levels of coverage overall), the West (66%) and the Northeast (43%) had higher levels, compared to all other regions (<12%) (Table 1 and Fig. 1). The Northeast had significantly higher coverage at school grounds (51%), compared to other regions (*p* < 0.001).

Visually, there were clear geographic patterns in smokefree outdoor space policies, whereby there appeared to be generally low consistency at the state-level (Fig. 1). Clusters of jurisdictions which adopted policies restricting smoking on both school grounds and playgrounds can be observed, particularly in Mississippi, New Jersey, Colorado, and Oregon. New York is the only state covered for both types of outdoor areas. Only 10 states had a state policy on *either* playgrounds or school grounds.

In the mixed effects logistic regression model (Table 2), all independent variables and the interaction term were found to be significant predictors of whether a smokefree playgrounds policy had been adopted. We observed a clear wealth gradient whereby odds increased with wealth (, after adjustment for other covariates. The highest income group had more than three times the odds of having a policy, compared to the lowest income group (OR 3.44, 95%CI: 2.08–4.40). We found small, significant effects for both percentage of the population without a high school diploma and percentage white, whereby higher percentages were associated with lower odds of having a policy. We also found that the effect of high ethnic heterogeneity differed significantly by region, but was consistently positively associated with having a policy (*p* < 0.001). For example, the ratio of odds of having a policy in zip codes with high versus low heterogeneity was highest in the Midwest (OR 3.00), followed by the Southwest (OR 2.34), the West (2.08), the Northeast (OR 1.99), the Southeast (OR 1.31) and no real difference in Alaska/Hawaii (OR ~ 1.00).

**Table 2:** Hierarchical logistic regression model results

Predicting the presence of a smokefree policy for playgrounds		OR	95% CI		p*
	High Heterogeneity	**2.08**	1.40	3.10	< 0.001
	Percent White	**0.99**	0.98	0.99	< 0.001
Income Quantiles	1 - Lowest	ref			< 0.001
	2	**1.27**	1.01	1.60	
	3	**1.70**	1.35	2.15	
	4 - Highest	**3.44**	2.68	4.40	
Educational Attainment	% without a High School Diploma	**0.98**	0.97	0.99	< 0.001
Region	West	ref			0.043
	AK/HI	**< 0.001**	< 0.001	< 0.001	
	Midwest	**0.09**	0.01	1.37	
	Southwest	**0.04**	0.001	1.87	
	Northeast	**4.15**	0.27	63.07	
	Southeast	**0.52**	0.04	7.57	
Diversity X Region	High Heterogeneity. West	ref			< 0.001
	High Heterogeneity. AK/HI	**< 0.001**	< 0.001	< 0.001	
	High Heterogeneity. Midwest	**1.98**	1.22	3.19	
	High Heterogeneity. Southwest	**1.12**	0.48	2.63	
	High Heterogeneity. Northeast	**0.95**	0.59	1.52	
	High Heterogeneity. Southeast	**0.63**	0.39	1.01	
	Constant	**0.08**	0.01	0.65	0.018

*Overall *p*-values for categorical variables calculated using likelihood ratio test

Bold indicates significance at the *p* < 0.05 level

## Discussion

Our findings confirm that there are stark differences in smokefree outdoor space policy coverage in places frequented by children based on ethnicity, socioeconomic status, educational attainment, and region. A serious lack of smokefree policy protection exists for children in the United States; only 8% of children in the United States live in zip codes with both smokefree playgrounds and school grounds. This low joint coverage is regardless of ethnicity.

It is important to eliminate exposure to tobacco use for children for three main reasons. Reducing exposure to tobacco use serves to reduce both the social normality and acceptability of smoking, as well as exposure to SHS. Furthermore, ethnic inequalities in coverage of playground policies may contribute to long-term health inequalities. The differences in the coverage spread for playgrounds (where there was a wide coverage variance by ethnicity) and school grounds (where there was a much narrower spread) may be related to different authorities covering the different types of child-related areas. Although playground and school ground policies are comparatively rare, these policies have been supported by 76% or more of the public in surveys within the USA since 2005 [36].

Regional differences in the coverage of smokefree space policies were large – and the effect of ethnic heterogeneity varied. Virtually no coverage for both school grounds and playgrounds was observed in Alaska/Hawaii and the Southwest, with all other regions below 3%, except for the Northeast. Considering the importance of smokefree outdoor space policy in reductions in tobacco prevalence and normality, it is crucial to address regional differences in order to continue to see equitable declines in tobacco use. These large regional discrepancies help to explain some of the differences in coverage by ethnicity. There is a large discrepancy between the Northeast and the rest of the United States, in the coverage for both playgrounds and school grounds, which warrants further study. The Northeast had coverage at a rate of 39% (largely due to New York State), compared to less than 3% for the rest of the country.

The effect of ethnic heterogeneity was significantly different by region, but was consistent in that more heterogeneous zip codes had higher odds of coverage in every region. The Midwest showed the largest effect of ethnic heterogeneity, with diverse zip codes over three times more likely to have a smokefree playgrounds policy.

This research found evidence of inequalities in smokefree outdoor space policy coverage by socioeconomic status and by educational attainment. In particular, the odds of having a policy covering playgrounds was found to decrease as the proportion of the population without a high school diploma increased, and as the average income of the zip code decreased. These differences illustrate ongoing shortages in protection for the United States’ poorest and least educated citizens, which begin at birth and impact the likelihood of smoking and quitting.

### Research implications

Considering the inequities observed in this study, the forces which impact policy adoption are an important next step to research. Successful adoption of policies is likely due in part to differing political climates between areas, as well as efforts by both the tobacco industry and by smokefree advocates [37–39]. Our speculation is that smokefree advocate organizations have a greater relative impact in cities compared to tobacco marketing. Urban areas are known to lean towards voting for Democrats, have more robust public health systems, and generally be more accepting of government oversight and legislature [32, 40, 41]. Future research could explore relationships between population density and policy adoption.

### Policy implications

In order to further declines in smoking and uptake among youth, policies must address the low level of coverage in places frequented by children. Considering the impact these policies have on the perceptions of smoking which children develop, as well as the low existing level of coverage nationally, policies aimed at protecting children in school grounds and playgrounds may have a large impact on smoking trends, now and into the future.

## Conclusion

This research revealed clear inequalities in the coverage of smokefree outdoor space policies, specifically ethnic, income, educational and regional inequalities. Inequalities in the banning of smoking in areas frequented by children may perpetuate the normality of smoking within subgroups of the United States’ population, and thus reinforce disparities in the negative impacts of exposure to smoking. Our regression results indicate that income inequalities between places may influence smokefree policies, and that policy coverage differences have real potential to exacerbate existing health inequalities. Legislative coverage of smokefree outdoor spaces is an important, cost-effective option to protect adults and children from SHS exposure and the normality of smoking. But, if not implemented equitably, tobacco-related health inequities may persist.
